# 

**Published:** 1878-08

**Authors:** 


THE BUFFALO

### MEDICAL AND SURGICAL JOURNAL

400

VOL. XVIII. AUGUST, 1878. NO. 1.

